# What Is the Role of Imaging in Cancers?

**DOI:** 10.3390/cancers12061494

**Published:** 2020-06-08

**Authors:** Laura Evangelista, Stefano Fanti

**Affiliations:** 1 Nuclear Medicine Unit, Department of Medicine (DIMED), University of Padua, 35128 Padua, Italy; 2 Department of Nuclear Medicine, Sant’Orsola-Malpighi Hospital, University of Bologna, 40138 Bologna, Italy; stefano.fanti@aosp.bo.it

In the issue entitled “Role of Medical Imaging in Cancers”, 33 papers have been collected (23 original articles, 8 reviews, 1 brief report and 1 perspective). All the papers focus on different topics, mainly on the role of positron emission tomography (PET) imaging in the management of oncological patients. 

Table 1 shows a summary of the topics and the papers included for each topic [1,2,3,4,5,6,7,8,9,10,11,12,13,14,15,16,17,18,19,20,21,22,23,24,25,26,27,28,29,30,31,32,33]. The majority of papers are focused on prostate cancer (PCa) and radiomics. The first topic has continuously gained interest in last years, in particular after the introduction of prostate specific membrane antigen (PSMA)-based radiopharmaceuticals, as extensively reported by Hoffmann et al. [18] in a cohort of more than 580 patients with recurrent PCa, showing that, after radical prostatectomy, PSMA PET/computed tomography (CT) was able to detect the presence of recurrent disease in more than 50% of patients with a PSA level < 1.24 ng/mL. Moreover, Treglia et al. [24], in a meta-analysis, clearly demonstrated the high detection rate of 18F-labeled PSMA also in patients with a PSA levels < 0.5 ng/mL. Nevertheless, Fanti et al. [20] underlined that, although there is a large amount of data on PSMA PET in PCa, the diagnostic procedures are still underutilized in clinical practice. The role of imaging in PCa was also analyzed by using different radiopharmaceutical agents and specific software. Laudicella et al. [23] tested the utility of 18F-FACBC (or fluciclovine) in recurrent PCa, showing a high diagnostic performance for the detection of local recurrence, mainly in the prostatic fossa. Bauckneht et al. [19] support the role of 18F-Fluorodeoxyglucose (FDG) PET/CT as a tool for patient selection and response assessment in metastatic castrate resistant PCa patients undergoing 223Ra administration. Furthermore, in this latter setting of patients, a segmentation-based tumor load at 99mTc-dysphonate SPECT/CT was linked with clinical outcome [21]. Finally, a radiomic approach with a specific magnetic resonance imaging (MRI) protocol can be useful to appropriately detect and characterize PCa [31]. Radiomics is an emerging field, defined as the extraction of quantitative data from medical images by using specific software. It can be applied to all medical imaging, such as CT, MRI, PET/CT or PET/MRI. In the last years, a large amount of data has been published, such as in the current issue of *Cancers*, in order to apply this in diverse settings of disease. Castaldo et al. [30] and Schiano et al. [32] evaluated the role of radiomics in breast cancer. The first group of authors concluded the ability of radiomic features by MRI to discriminate major breast cancer molecular subtypes, thus potentially guiding a personalized treatment [30]. The second group by Schiano et al. [32] showed that the combination of radiomic features by FDG PET/MRI and molecular data are able to predict the synchronous metastatic disease more accurately than a single information. Fujima et al. [33] found a correlation between the clinical outcome and machine-learning algorithm using various MRI-derived data in patients with sinonasal squamous cell carcinomas.

Indeed, radiomics can be used as a tool for the prediction of outcomes in terms of response to therapy or prognosis in patients undergoing immune check point inhibitors, as stated by Polverari et al. [29]. The use of imaging as a predictive biomarker of response to immunotherapy has been discussed by some authors [5,6,7], in the present issue. Both in the reviews and in the original articles, nuclear medicine images can be considered as a non-invasive method that is able to predict the response to immunotherapy, mainly in combination with other clinical or biological markers. Moreover, FDG PET/CT can be helpful in predicting the response to therapy in patients with head and neck squamous cell carcinoma, due to the high correlation between micro-vessel density and FDG uptake in terms of SUVmax [28]. This latter semiquantitative parameter is predictive of overall survival in patients with recurrent ovarian cancer [25]. The same author [27] reported that PET/CT can predict more accurately than MRI the response to concurrent chemoradiation treatment in T2 cervical cancer patients. Finally, from a German experience in a small cohort of patients (*n* = 16) affected by high-risk soft tissue sarcoma, the authors of [26] found that FDG kinetic analysis can be considered as a marker of response to pazopanib in soft-tissue sarcoma, and therefore it could guide anti-angiogenetic therapy. 

The residual papers focused on different topics by analyzing diverse imaging modalities from preclinical [17], to gold nanoparticles for CT imaging [3,4], to specific pathologies (i.e., breast cancer [1,2] or pancreas [13,14] or meningioma [10]), to multicenter trials [8,22]. The common conclusion of these contributions is the current and future support that medical imaging can add to clinical information. The continuous developments of new radiopharmaceutical agents or the production of new technologies (i.e., scanners or software) will lead to increasingly personalized medicine. 

## Figures and Tables

**Table 1:** Summary of articles in the special issue.

Topic (in Alphabetic Order)	Type of Paper	
	Original Articles (ref)	Reviews or Brief Article or Perspectives (ref)
Breast cancer	None	Salvatore et al. [1], Hildebrandt et al. [2]
CT imaging	Kimm et al. [3], Lee et al. [4]	None
Immunotherapy	Castello et al. [5]	Frega et al. [6], Decarez et al. [7]
Lymphoma	Albano et al. [8]	Voltin et al. [9]
Meningioma	None	Laudicella et al. [10]
MRI	Jin et al. [11], Usuda et al. [12]	None
Pancreas	None	Serafini et al. [13], Montemagno et al. [14]
PET/MRI	Samolyk-Kogaczewska et al. [15], Incoronato et al. [16]	None
Preclinical	Montemagno et al. [17]	None
Prostate and genito-urinary	Hoffmann et al. [18], Bauckneht et al. [19], Fanti et al. [20], Fiz et al. [21], Zattoni et al. [22]	Laudicella et al. [23], Treglia et al. [24]
Response to therapy or predictors	Perrone et al. [25], Sachpekidis et al. [26], Perrone et al. [27], Surov et al. [28]	None
Radiomics	Polverari et al. [29], Castaldo et al. [30], Monti et al. [31], Schiano et al. [32], Fujima et al. [33]	None
